# Alleviating salinity stress in canola (*Brassica napus* L.) through exogenous application of salicylic acid

**DOI:** 10.1186/s12870-024-05314-y

**Published:** 2024-06-27

**Authors:** Maria Ilyas, Muhammad Faisal Maqsood, Muhammad Shahbaz, Usman Zulfiqar, Kamran Ahmad, Nargis Naz, Muhammad Fraz Ali, Muhammad Ahmad, Qasim Ali, Jean Wan Hong Yong, Hayssam M. Ali

**Affiliations:** 1 Department of Botany, The Islamia University of Bahawalpur, Bahawalpur, 63100 Pakistan; 2 Department of Botany, University of Agriculture, Faisalabad, 38040 Pakistan; 3 Department of Agronomy, Faculty of Agriculture and Environment, The Islamia University of Bahawalpur, Bahawalpur, 63100 Pakistan; 4 Department of Botany, College of Life Sciences, Northwest A&F University, Yangling , Shaanxi, 712100 China; 5 College of Agronomy, Northwest A&F University, Yangling, Xianyang, 712100 China; 6 Department of Agronomy, University of Agriculture, Faisalabad, 38040 Pakistan; 7 Department of Soil Science, Faculty of Agriculture and Environment, The Islamia University of Bahawalpur, Bahawalpur, 63100 Pakistan; 8 Department of Biosystems and Technology, Swedish University of Agricultural Sciences, Alnarp, Sweden; 9 Department of Botany and Microbiology, College of Science, King Saud University, 11451 Riyadh, Saudi Arabia

**Keywords:** Canola, Salinity stress, Salicylic acid, Antioxidants, Osmolytes

## Abstract

Canola, a vital oilseed crop, is grown globally for food and biodiesel. With the enormous demand for growing various crops, the utilization of agriculturally marginal lands is emerging as an attractive alternative, including brackish-saline transitional lands. Salinity is a major abiotic stress limiting growth and productivity of most crops, and causing food insecurity. Salicylic acid (SA), a small-molecule phenolic compound, is an essential plant defense phytohormone that promotes immunity against pathogens. Recently, several studies have reported that SA was able to improve plant resilience to withstand high salinity. For this purpose, a pot experiment was carried out to ameliorate the negative effects of sodium chloride (NaCl) on canola plants through foliar application of SA. Two canola varieties Faisal (V1) and Super (V2) were assessed for their growth performance during exposure to high salinity i.e. 0 mM NaCl (control) and 200 mM NaCl. Three levels of SA (0, 10, and 20 mM) were applied through foliar spray. The experimental design used for this study was completely randomized design (CRD) with three replicates. The salt stress reduced the shoot and root fresh weights up to 50.3% and 47% respectively. In addition, foliar chlorophyll *a* and *b* contents decreased up to 61–65%. Meanwhile, SA treatment diminished the negative effects of salinity and enhanced the shoot fresh weight (49.5%), root dry weight (70%), chl. *a* (36%) and chl. *b* (67%). Plants treated with SA showed an increased levels of both enzymatic *i.e. *(superoxide dismutase (27%), peroxidase (16%) and catalase (34%)) and non-enzymatic antioxidants *i.e.* total soluble protein (20%), total soluble sugar (17%), total phenolic (22%) flavonoids (19%), anthocyanin (23%), and endogenous ascorbic acid (23%). Application of SA also increased the levels of osmolytes *i.e.* glycine betaine (31%) and total free proline (24%). Salinity increased the concentration of Na^+^ ions and concomitantly decreased the K^+^ and Ca^2+^ absorption in canola plants. Overall, the foliar treatments of SA were quite effective in reducing the negative effects of salinity. By comparing both varieties of canola, it was observed that variety V2 (Super) grew better than variety V1 (Faisal). Interestingly, 20 mM foliar application of SA proved to be effective in ameliorating the negative effects of high salinity in canola plants.

## Introduction

With the enormous demand for growing various crops and the rising controversies regarding the competition between food and energy crops for agricultural land, the utilization of agriculturally marginal lands is emerging as an attractive alternative, including salt-affected wastelands and brackish-saline transitional lands [1, 2]. Salinity stress poses a significant threat to crop yields and especially in arid and semiarid regions globally, with a 10% annual increase. Projections indicate that by the mid-twenty-first century, 50% of arable land could be lost due to increasing salinity, emphasizing the urgent need for effective mitigation strategies [3–5]. Currently, high soil salinity is causing significant disruptions to agricultural production on a global scale [6]. Salt stress significantly decreases crop yields on infertile and partially fertile lands, leading to a reduction of over 50% in standard yields. This reduction in crop productivity is primarily attributed to the disruption of the plants' nutritional and water balance [7–10]. The ability of plants to withstand high salt levels is an intricate process, involving various factors such as morphological, physiological, and biochemical mechanisms [8, 10, 11]. Sodium (Na^+^) and chloride (Cl^−^) ions create a high osmotic potential, leading to inadequate water and nutrient supply to roots and causing cellular disruptions [8, 12, 13].

High salinity exerts a profound negative growth effects on canola (*Brassica napus* L.) leading to smaller plants and yield [6, 14]. This, along with osmotic stress, collectively impairs the growth, development and overall survival of the plant uptake and homeostasis in plant body [15, 16]. Mineral imbalances, such as excessive Na^+^ accumulation at toxic levels, can disrupt the normal metabolic processes within a plant's body. These imbalances lead to an elevated generation of ROS. In reaction to increased ROS production, plants activate a range of defense mechanisms, including the synthesis of both enzymatic and non-enzymatic antioxidants [9, 14, 17]. Exposure to salt stress results in increased activity of ROS scavenging enzymes such, as peroxidase, in plants. This heightened enzyme activity leads to greater lignification of plant tissues, ultimately restraining the growth of the plant [3, 18, 19].

Canola, a member of the Brassicaceae family, is an important and extensively grown crop globally. It holds the second position, following soybean, in the cultivation of oilseed crops for human consumption of edible oil and animal feed in the world [20]. Although widely recognized as a salt-resistant plant, its productivity and yield are lowered under high salinity conditions [21]. Additionally, it was reported that canola cultivars resistant to salt stress differ genotypically [22]. Canola oil is a versatile ingredient used in salad oils, salad dressings, and margarines, and it also plays a role in creating organogels. Defatted canola, on the other hand, has a wide range of food applications, including emulsifying, gelling, absorbing, stabilizing, thickening, forming oleo gels, and enhancing texture. It is important to note that while there are potential health benefits linked to canola protein, further long-term human studies are required to comprehensively validate these advantages [23]. Considering the detrimental effects of salinity on canola, such as an increase in sodium content and oxidative damage and a decrease in potassium uptake and seed yield [24]. It is imperative to explore strategies aimed at mitigating the negative effects of salt-related damage on the physiological characteristics and crop yield of canola.

In tandem with the availability of resources and environmental cues, plant ontogenic development is coordinated and carefully regulated by various endogenous growth regulators commonly known as phytohormones or biostimulants [25–27]. These phytohormones play crucial role in regulating various physiological and biochemical processes that govern plant responses under optimal and stress conditions [13, 28]. The involvement of cytokinins, gibberellins, auxins, abscisic acid, ethylene, strigolactones and brassinosteroids in growth and development has been well documented [13, 25, 29–32]. Recently, various studies have highlighted the role of phytohormones such as salicylic acid (SA), and jasmonates in the plant responses toward abiotic stresses [27, 29]. Specifically, the phytohormone SA is a phenolic compound that controls plant growth and development in both favorable and challenging environments [29, 33]. More recently, it was discovered that SA offers biological protection to plants against abiotic stresses. This protective function was attributed to its regulation of several essential physiological processes, such as photosynthesis, proline metabolism, nitrogen metabolism, glycine betaine biosynthesis, antioxidant mechanisms, and the overall water status of the plant. Consequently, SA is implicated in enhancing a plant's resistance to a range of abiotic stressors, including ozone, metal exposure, UV-B radiation, extreme temperatures, drought, and high salinity [25, 34]. Salicylic acid functions as a signal sensor in plants, regulating their responses and protecting cells from harmful effects like ion accumulation and cell death. It facilitates important processes such as antioxidant defense, nitrogen metabolism, photosynthesis, and coping with water stress. SA levels vary significantly among plant species and in response to environmental challenges [35]. The impact of naturally occurring SA levels in plants is associated with their developmental stage and exposure to external stimuli [36]. Additionally, applying exogenous SA treatment can enhance a plant's resilience to various stressors, such as salt, drought, heat, cold, and heavy metals [37, 38]. Under abiotic stress conditions, plants can trigger a sequence of gene expressions, some of which are associated with SA-dependent activation. These genes influence a range of biological processes, such as the production of molecular chaperones, antioxidants, and secondary metabolites [8, 11, 39].

Previous literature has indicated that the application of SA significantly enhances stress resistance in various crops under both saline and non-saline conditions. It was hypothesized that SA can alleviate the negative effects of salt stress on canola. This treatment was expected to enhance canola growth, photosynthetic pigment levels, antioxidant defense systems, and nutrient absorption under saline and non-saline conditions. Based on the hypothesis, the objective of present study was to investigate the effects of SA in optimizing the growth, photosynthetic activity, and antioxidant defense system of canola during growth in high salinity conditions.

## Materials and methods

### Experimental setup

An experiment was conducted at the Botanical Garden, The Islamia University of Bahawalpur, to investigate the effects of salinity on canola (*Brassica napus* L.) through exogenous salicylic acid (SA) application. This study was carried out from November-2022 to February-2023. Two canola varieties, namely Faisal (V1) and Super (V2), were exposed to two levels of salinity i.e. control and 200 mM NaCl. Salicylic acid was applied in three different concentrations (0, 10, and 20 mM) via foliar spray. Plastic pots with 8 kg of soil were utilized for the growth experiment. In each pot, fifteen seeds were sown, and after two weeks of germination, seven plants were maintained after thinning. Salinity was applied with regular intervals in the form of a solution after 45 days of sowing. A foliar spray of SA was applied after two weeks of salinity application. After three weeks of SA application, data related to various growth parameters, i.e., chlorophyll pigments, biochemical characteristics, and antioxidant profiles were recorded. The treatments were applied in the following order; T0 = Control + 0 mM SA, T1 = Control + 10 mM SA, T2 = Control + 20 mM SA, T3 = 200 mM NaCl + 0 mM SA, T4 = 200 mM NaCl + 10 mM SA and T5 = 200 mM NaCl + 20 mM SA. All the experiments were done in compliance with relevant institutional, national, and international guidelines and legislation. High research standards were maintained throughout the experiments and following the various established scientific protocols [13, 28, 40, 41].

### Soil analysis

Soil analysis was done at the Regional Agricultural Research Institute, Bahawalpur. Soil sample were obtained at 15 cm depth for analysis. The soil pH was 7.98 and electrical conductivity was 0.29 mS cm^−1^. In addition to these physical attributes, organic matter (0.63%), available phosphorus (32 mg kg^−1^), available potassium (28 mg kg^−1^), saturated percentage (28%) and texture (sandy loamy) were also examined.

### Morphological parameters

Shoot and root lengths were measured using a measuring scale. Additionally, the fresh weight of plant samples, comprising both shoots and roots, was promptly determined upon harvesting using a digital weighing balance. Subsequently, the samples underwent oven drying within a temperature range of 65 °C. After two weeks of drying, the dry weights of both shoots and roots were measured using a digital balance.

### Photosynthetic pigments

For determination of chlorophyll contents, 0.1 g leaf sample were ground in 5 ml of 80% acetone. Samples were kept overnight and absorbance for each sample was recorded at 663, 645, and 480 nm with spectrophotometer [42].

### Reactive oxygen species (ROS)

#### Hydrogen peroxide (H_2_O_2_)

The technique described by Velikova et al*.* [43]*,* was employed to measure hydrogen peroxide levels. Initially, 0.25 g of leaf sample was ground in 2 ml of 0.1% TCA under chilled conditions. Following centrifugation at 1500 rpm for 20 min, the supernatant was isolated. Subsequently, a test tube was filled with 0.5 mL of phosphate buffer, 0.5 ml of leaf sample, and 1 ml of potassium iodide solution (165.9 g potassium iodide in 1 L of distilled water). After careful vortexing, the absorbance was recorded at 390 nm using a spectrophotometer.

#### Malondialdehyde (MDA)

Malondialdehyde levels were determined according to Yagi (1982). Initially, 0.25 g of ground leaf sample was added to 2 ml of 0.1% TCA solution (0.1 g TCA in 100 ml of distilled water). The supernatant was separated after centrifuging at 1500 rpm for 20 min. A solution was prepared by dissolving 20 g of TCA and 0.5 g of TBA in 100 ml of distilled water. In a test tube, 4 ml of the solution and 1 ml of the supernatant were added. The solution was then placed in a water bath at 95ºC for 30 min. After removal from the water bath, it was allowed to cool, and readings were taken at 532 and 600 nm.

### Enzymatic antioxidants activities

#### Catalase (CAT)

The Chance and Maehly [44], method was employed to measure CAT activity. Initially, 5 ml of phosphate buffer was added to 0.2 g of ground leaf sample. The solution was then centrifuged at 1500 rpm for 20 min, followed by the perpendicular separation of the supernatant. Subsequently, a cuvette was filled with 0.1 ml of the sample, 1 ml of H_2_O_2_, and 1.9 ml of phosphate buffer. At intervals of 0, 30, 60, and 90 s, the absorbance was measured at 240 nm using a spectrophotometer.

#### Superoxide dismutase (SOD)

Superoxide dismutase activity was measured using the method described by Giannopolitis and Ries [45]. Reaction mixture contains 50 µL of nitroblue tetrozolium (NBT), 50 µL of riboflavin, 100 µL of L-methionine, 250 µL of phosphate buffer, 100 µL of tritox and 150 µL of distilled water. The sample was exposed to light for 20 min, and the absorbance was recorded at 560 nm using a spectrophotometer.

#### Peroxidase (POD)

The Chance and Maehly [44], method was employed to measure POD activity. A cuvette was prepared with 0.05 ml sample extract, 7.5 ml phosphate buffer, 0.1 ml guaicol solution (335 µl H_2_O_2_ + 15 µl phosphate buffer), and 0.1 ml H_2_O_2_ solution (100 µl H_2_O_2_ + 20 µl phosphate buffer). The absorbance was recorded at 470 nm with spectrophotometer at 0, 30, 60, and 90-s intervals.

### Non-Enzymatic antioxidants activities

#### Total phenolics

The amount of total phenolics was calculated in accordance with Julkenen-Titto [46]. Leaf material (0.5 g) was extracted using 10 mL of 80% acetone. One milliliter of the supernatant was mixed with 5 mL of 20% Na_2_CO_3_ and 1 mL of Folin-Ciocalteu phenol reagent. Distilled water was added to bring the total volume of the mixture to 10 mL. The absorbance of reaction mixture was recorded at 750 nm with spectrophotometer.

#### Flavonoids

According to Marinova et al., [47], flavonoid contents were measured. A brief incubation at 25 °C was followed by the addition of 1 mL of the ethanol extract to 300 L of NaNO_3_. Then, AIC1_3_ (300 µL) was added, and the mixture was left at room temperature for 5 min. The mixture was further enhanced with 2 mL of NaOH (1 M), which was allowed to cool at room temperature for 10 min. The mixture's volume was increased to 10 mL using distilled water. The absorbance was observed at 510 nm through spectrophotometer.

#### Ascorbic acid (AsA)

The amount of endogenous AsA was calculated according to Mukherjee and Choudhuri [48], protocol. For extraction, 0.25 g fresh leaf sample was crushed in 5 mL of 6% trichloroacetic acid. 4 mL of the extract, 2 mL of 2% dinitrophenyl hydrazine in acidic medium and a drop of thiourea in 70% ethanol were added. The mixture was heated in a water bath for 15 min and then chilled to room temperature. After cooling, 5 mL of 80% H_2_SO_4_ was added to the solution, which was then maintained on ice at 0 °C. Absorbance was recorded at 530 nm with spectrophotometer.

#### Total soluble sugars (TSS)

To measure the total soluble sugar, 0.5 g of fresh leaf material was extracted using 80% ethanol. 100 mL of ethanol extract were combined with 3 mL of enthrone reagent, which had been previously prepared in 72% sulfuric acid. The mixture was then heated at 95 °C for 15 min. The reaction mixture was allowed to cool at room temperature for 30 min. The absorbance of the mixture was measured at 620 nm using a spectrophotometer [49].

#### Total soluble proteins (TSP)

Bradford reagent was prepared to measure the total soluble proteins. This reagent was made by mixing 1 L of distilled water with 100 ml of 85% phosphoric acid, 0.1 g of brilliant blue, and 50 ml of 95% ethanol. The freshly made reagent was filtered using filter paper three to four times. Each test tube contained 5 ml of reagent and 0.1 ml of leaf sample Absorbance was recorded at 595 nm by using a spectrophotometer.

#### Anthocyanin

For the measurement of anthocyanin, in 0.2 g of crushed leaf sample was mixed with 5 ml of acidified methanol. Acidified methanol was prepared by mixing 120 mL of methanol with 1 mL of HCl. The samples were placed in appropriately labeled test tubes, which were then transferred to a water bath at 50 °C for one hour. Afterward, the test tube were removed, and the absorbance were recorded at 535 nm using a spectrophotometer [50].

#### Total free proline

Proline content was measured by crushing 0.25 g of fresh leaf material in 5 ml of 3% sulfosalicylic acid, followed by filtering the extract. One milliliter filtrate was retained in a test tube containing 1 ml of acid ninhydrin and 1 ml of glacial acetic acid, and then heated in a water bath for 90 min at 100 °C. The vortexing created two layers, and a spectrophotometer was used to measure the absorbance of the upper pinkish layer at 520 nm.

#### *Glycine* betaine (GB)

For glycine betaine determination, 0.25 g of fresh material was extracted in 5 ml of distilled water. The extract was centrifuged at 12,000 rpm for 15 min. 500 µl of the resulting extract were added to a test tube along with 1 ml of 2 N H_2_SO_4_ and 1 ml of the sample extract. After adding 0.2 ml of potassium tri-iodide, the test tubes were chilled for 90 min. To the ice-cooled test tubes, distilled water and 6 ml of 1, 2-dichloroethane were added, respectively. Two distinct layers were created, and the lower layer was used for measuring the absorbance at 365 nm with a spectrophotometer.

#### Ion analysis of root and shoot (Na^+^, K^+^, Ca^2+^)

Oven-dried samples (0.1 g) of the root and shoot were kept in distinct, labelled conical flasks. Following which, 5 ml of pure H_2_SO_4_ were added to each flask and covered overnight. The next day, the flasks were placed on a hot griddle, and H_2_O_2_ was gradually added while heating the sample until the solution become transparent. After cooling, the solution was filtered using filter paper, and distilled water was added to maintain the volume up to 50 mL. The levels of Na^+^, K^+^, and Ca^2+^ were then measured using the flame photometer.

### Statistical analysis

For statistical analysis, software (Statistic 8.1) was used. A three-way analysis of variance (ANOVA) was used to analyze the data. Microsoft Excel was used to create the graphs. Radar analysis and correlation matrix were performed by using R-studio version R-4.3.0 (R Development Core Team 2021).

## Results

### Morphological parameters

**Fig. 1:**
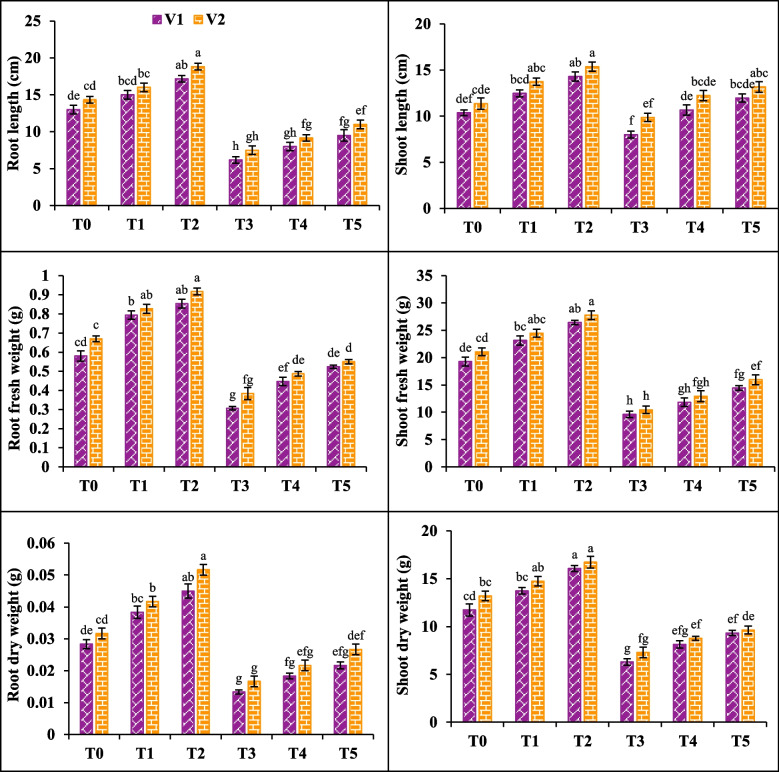
Effect of salicylic acid (SA) on root length, shoot length, root fresh weight, shoot fresh weight, root dry weight and shoot dry weight of canola under salt stress. The three replicates ± SE is shown by the error bars above the means. For a parameter, means that share same letter do not differ significantly at *p* < 0.05. (V1 = Faisal; V2 = Super; T0 = Control + 0 mM SA, T1 = Control + 10 mM SA, T2 = Control + 20 mM SA, T3 = 200 mM NaCl + 0 mM SA, T4 = 200 mM NaCl + 10 mM SA and T5 = 200 mM NaCl + 20 mM SA)

The application of 200 mM NaCl (T3) considerably affected the length of root and shoot, their fresh weights and dry weights in both varieties. A considerable reduction recorded in root length, fresh weight and dry weight (52, 47 and 52%) in V1 and (47, 42 and 47%) in V2. It is also observed that under T3 salt stress decreased the shoot length, fresh weight and dry weight in V1 (22, 50 and 40%) and V2 (13, 50 and 40%). However, exogenous application of SA (T5) increased the root length, fresh weights and dry weights in V1 54%, 70% and 62% and 31%, 36% and 63% respectively in V2. Similarly, foliar application of SA also enhanced the shoot length, fresh weight and dry weight in V1 (49, 49 and 100%) and V2 (35, 38 and 100%) respectively (Fig. 1).

### Photosynthetic pigments and ROS

**Fig. 2:**
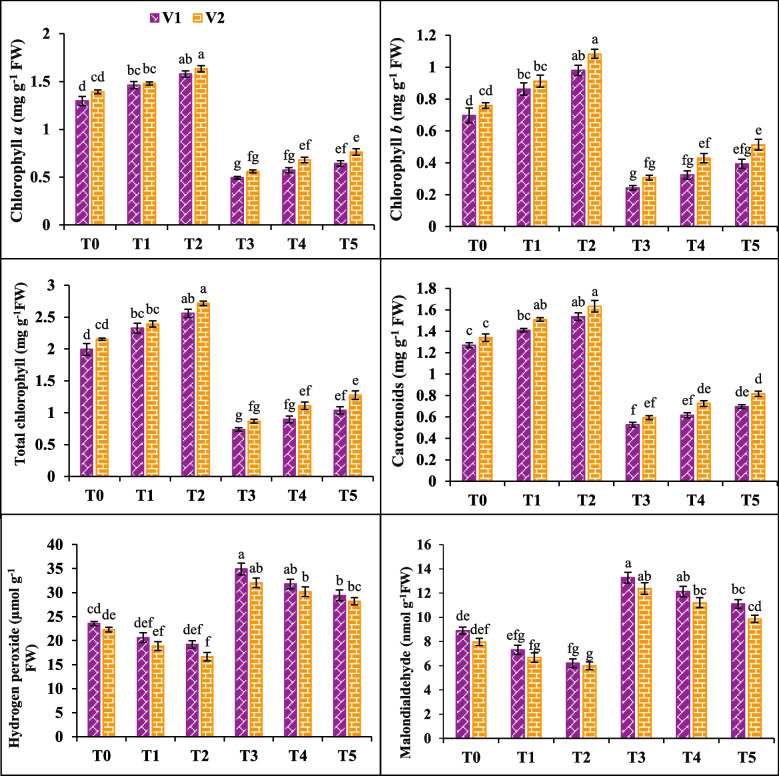
Effects of salicylic acid (SA) on chlorophyll a chlorophyll b total chlorophyll, carotenoids, hydrogen peroxide and malondialdehyde of canola under salt stress. The three replicates ± SE is shown by the error bars above the means. For a parameter, the means that share same letter do not differ significantly at *p* < 0.05. (V1 = Faisal; V2 = Super; T0 = Control + 0 mM SA, T1 = Control + 10 mM SA, T2 = Control + 20 mM SA, T3 = 200 mM NaCl + 0 mM SA, T4 = 200 mM NaCl + 10 mM SA and T5 = 200 mM NaCl + 20 mM SA)

The exposure of canola to 200 mM NaCl significantly altered the levels of photosynthetic pigments (chlorophyll a, chlorophyll b, total chlorophyll, carotenoids) and ROS (hydrogen peroxide and malondialdehyde) in both varieties. There was a notable decrease in chlorophyll a, chlorophyll b, total chlorophyll and carotenoids contents by 61, 65, 63 and 58% in V1 respectively; 59.8, 59.6, 59.7 and 55% in V2 under T3. Concomitantly, exposure to NaCl stress (T3) increased the production of ROS (H_2_O_2_, MDA) in V1 (48% and 49%) and V2 (43% and 55%). In contrast, SA (20 mM) application had a positive effect and increased the chlorophyll a, chlorophyll b, total chlorophyll and carotenoids in V1 (30, 61, 40 and 32%) and V2 (21, 42, 28 and 22%) under T2. Foliar application of 20 mM SA markedly reduced the hydrogen peroxide and malondialdehyde by 18%, and 30% in V1 and 25% and 24% in V2 (Fig. 2).

### SOD, POD, CAT, TSP, TSS and flavonoids

**Fig. 3:**
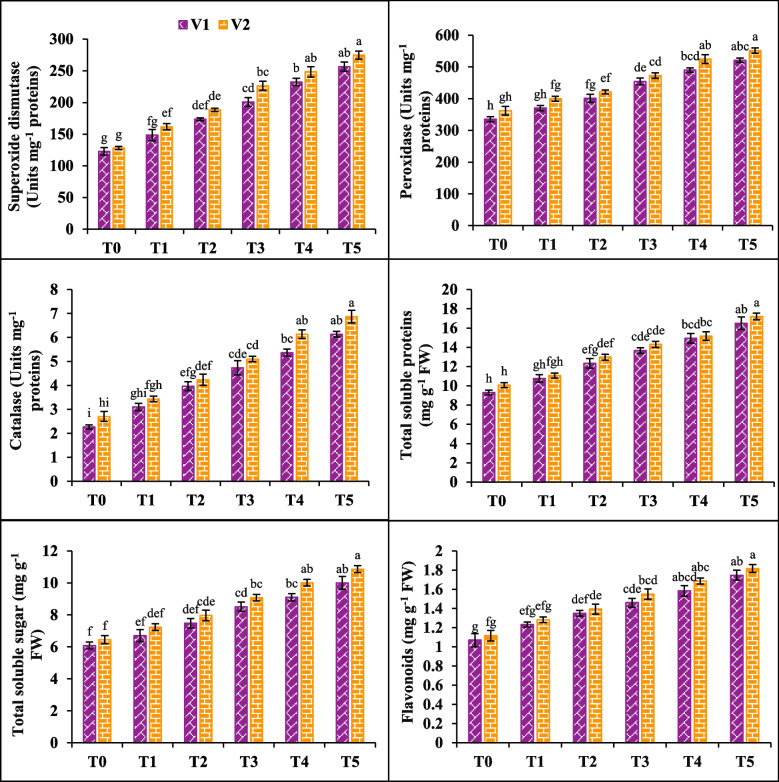
Effects of salicylic acid (SA) on superoxide dismutase (SOD), peroxidase (POD), catalase (CAT), total soluble proteins (TSP), total soluble sugars (TSS) and flavonoids of canola under salt stress. The three replicates ± SE is shown by the error bars above the means. For a parameter, means that share same letter do not differ significantly at *p* < 0.05. (V1 = Faisal; V2 = Super; T0 = Control + 0 mM SA, T1 = Control + 10 mM SA, T2 = Control + 20 mM SA, T3 = 200 mM NaCl + 0 mM SA, T4 = 200 mM NaCl + 10 mM SA and T5 = 200 mM NaCl + 20 mM SA)

High concentrations of NaCl (200 mM) had a significant impact on superoxidase (SOD), peroxidase (POD), catalase (CAT), total soluble proteins (TSP), total soluble sugars (TSS) and flavonoids in both plant varieties (V1 and V2). SOD, POD, CAT substantially increased (63, 35 and 108%), (76, 30 and 88%) in V1 and V2 respectively under stress conditions. Similarly, TSP, TSS and flavonoids slightly increased (46, 39 and 36%) in V1 and (42, 40 and 38%) V2 under saline conditions. However, the application of SA increased the SOD, POD, CAT in V1 (41, 19 and 75%) and V2 (47, 16 and 56%) under both stress and control conditions. SA (20 mM) also increased TSP, TSS and flavonoids by (32, 22 and 26%) in V1 while (28, 23 and 24%) in V2. Maximum increase recorded in V2 under T5 (Fig. 3).

### GB, proline, anthocyanin, ascorbic acid and phenolics

**Fig. 4:**
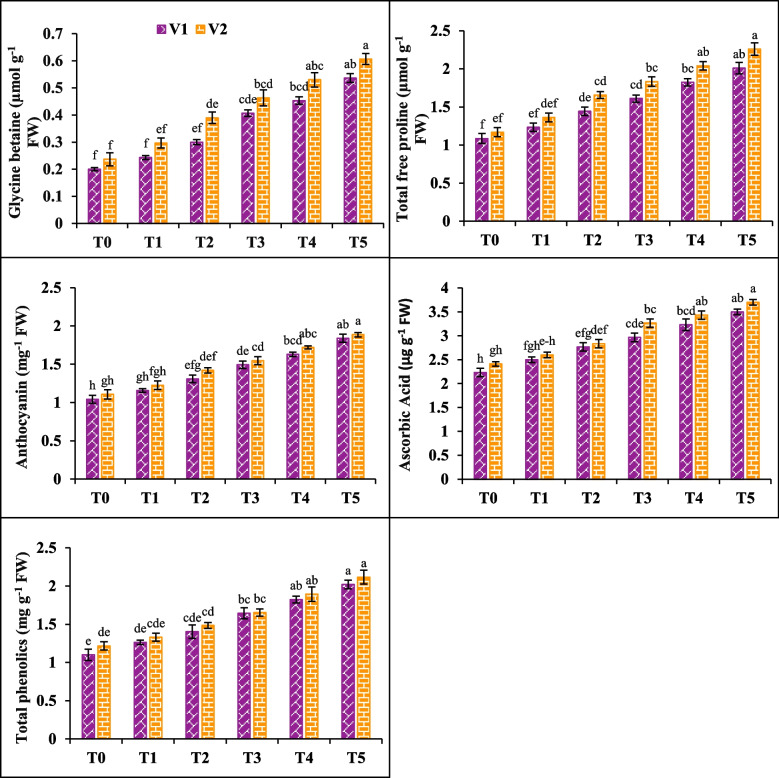
Effects of salicylic acid (SA) on glycine betaine, total free proline, anthocyanin, ascorbic acid and total phenolics of canola under salt stress. The three replicates ± SE is shown by the error bars above the means. For a parameter, the means that share same letter do not differ significantly at *p* < 0.05. (V1 = Faisal; V2 = Super; T0 = Control + 0 mM SA, T1 = Control + 10 mM SA, T2 = Control + 20 mM SA, T3 = 200 mM NaCl + 0 mM SA, T4 = 200 mM NaCl + 10 mM SA and T5 = 200 mM NaCl + 20 mM SA)

The levels of glycine betaine, proline, anthocyanin, ascorbic acid and phenolics increased after the application of 200 mM NaCl by 103, 48, 43, 32 and 49% in V1, whereas (95, 56, 39, 35 and 35%) in V2. Salicylic acid (20 mM) was applied foliarly that considerably enhanced the glycine betaine, proline, anthocyanin in V1 (50, 32 and 25%) and V2 (64, 41 and 28%). Similarly, ascorbic acid and phenolics also enhanced by (23% and 27%) (17% and 21%) in V1 and V2 respectively. The highest value recorded under T5 and lowest value seen under T0. Hence, V2 sowed better concentration as compared to V1 (Fig. 4).

### Root and shoot ions

**Fig. 5:**
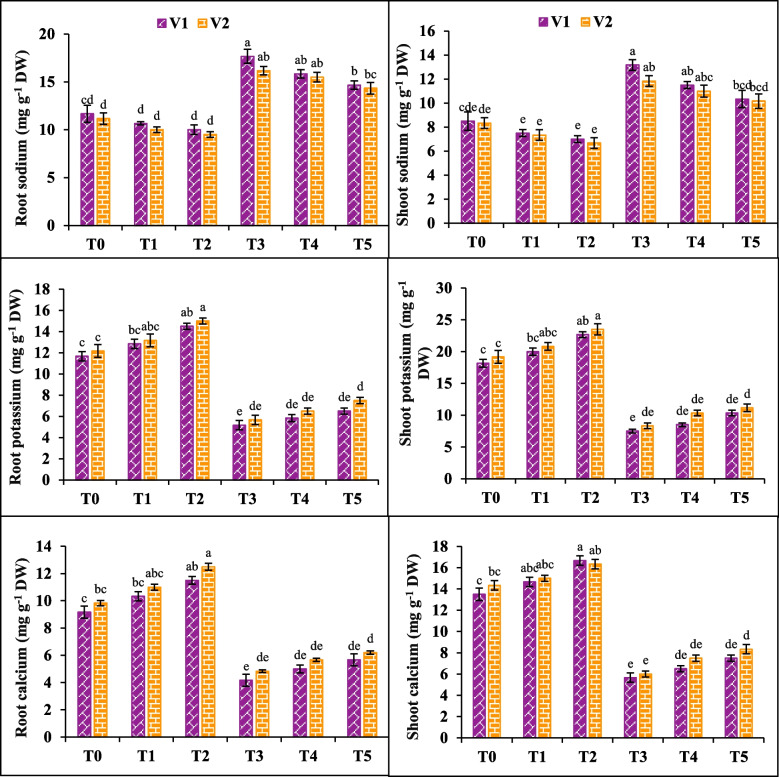
Effects of salicylic acid (SA) on root sodium, shoot sodium, root potassium, shoot potassium, root calcium and shoot calcium of canola under salt stress. The three replicates ± SE is shown by the error bars above the means. For a parameter, the means that share same letter do not differ significantly at *p* < 0.05. (V1 = Faisal; V2 = Super; T0 = Control + 0 mM SA, T1 = Control + 10 mM SA, T2 = Control + 20 mM SA, T3 = 200 mM NaCl + 0 mM SA, T4 = 200 mM NaCl + 10 mM SA and T5 = 200 mM NaCl + 20 mM SA)

High concentrations of NaCl (200 mM) gave significantly negative effects on root/shoot K^+^ and Ca^2+^ in both anola varieties (V1 and V2) by (55, 58, 54 and 58%) and (53, 56, 50 and 58%). On the other hand, application of NaCl enhanced the Na^+^ level in both root and shoot by (51% and 54%) in V1 and (44% and 42%) in V2. However, the negative effects of NaCl stress were mitigated by the application of SA (20 mM). Salicylic acid positively influenced on the root/shoot K^+^ and Ca^2+^ and increased the ion concentration in V1 (25, 37 36 and 32%) and V2 (23, 23, 27 and 25%), leading to decrease in root/shoot Na^+^ in both varieties (16, 21 and 14, 20%) respectively (Fig. 5).

### Radar analysis

**Fig. 6:**
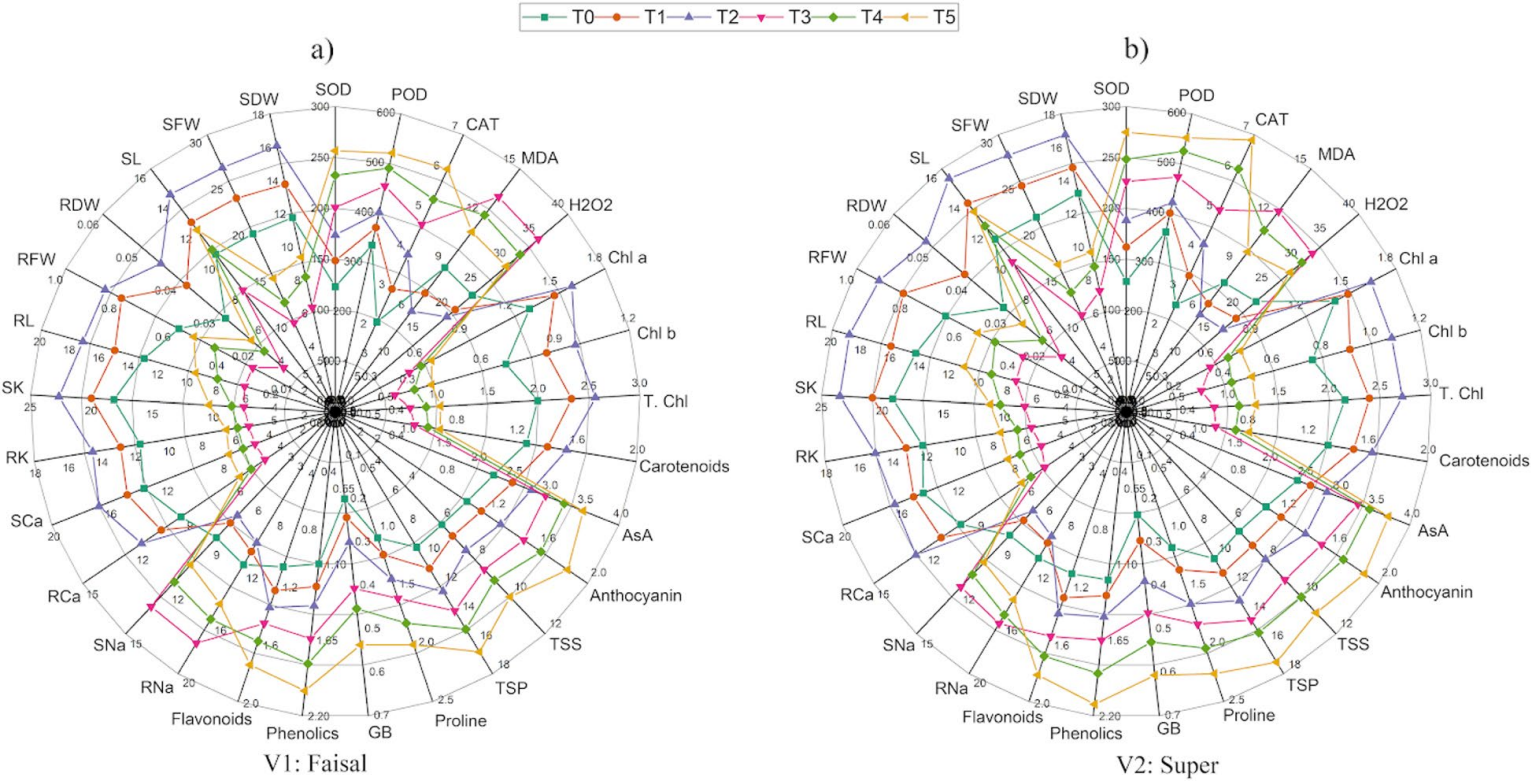
Radar chart showing the effects of salinity and salicylic acid on various parameters of canola varieties **a** Faisal **b** Super

The radar analysis presented the average observations of all parameters studied under salt stress, including morphological parameters, photosynthetic pigments, ROS, enzymatic antioxidants, non-enzymatic antioxidants, and salt ions (Fig. 6). According to the findings, the photosynthetic pigments of the canola plant increased under treatment T2, followed by T1 and control, which led to an increase in the morphological parameters of the plant, including the root and the shoot. The treatments T4 and T5 had the effect of reducing the photosynthetic pigments, while simultaneously increasing the activity of both enzymatic and non-enzymatic antioxidants. However, the morphological parameters of the root and shoot were reduced. The plant ions (Na^+^) for root and shoot have been increased under treatment T3, T4 and T5 while (Ca^2+^ and K^+^) have been increased under T1, T2 and control conditions.

**Fig. 7:**
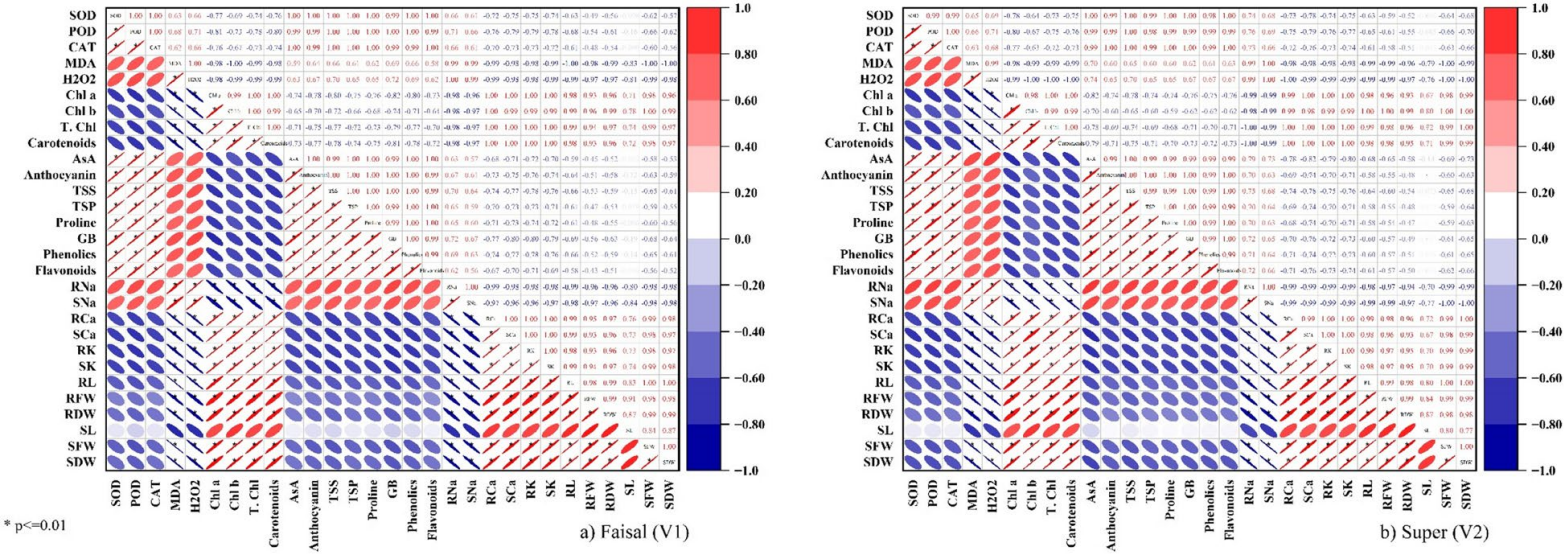
Correlation matrix of different parameters of canola varieties **a** Faisal **b** Super, and the different levels of salicylic acid (SA) under salt stress conditions

The correlation analysis revealed that the two types of canola studied traits had a similar correlation trend. Pearson’s correlation of antioxidants, non-enzymatic and biochemical traits with plant morphological parameters was analyzed for the two varieties of canola (Fig. 7). In canola plants, a highly positive correlation was observed between photosynthetic pigments (chlorophyll a, b, total chlorophyll, and carotenoids) with morphological parameters (SL, SFW, SDW RL, RFW, and RDW). Increasing these attributes directly correlated with the yield plant biomass and increased significantly (Fig. 7). A strong negative correlation was found between ROS (H_2_O_2_, MDA), enzymatic antioxidants (CAT, SOD, POD), non-enzymatic antioxidants (AsA, Anthocyanin, TSS, TSP, Proline, GB, Phenolics, Flavonoids), with morphological parameters of canola. Salt ions shows variation in correlation as Ca and K ions for root and shoot showed a positive correlation while the Na ion for root and shoot showed a negative correlation with morphological parameters of plants. The enzymatic antioxidants showed a significant strong correlation with non-enzymatic antioxidants (Fig. 7). Under salinity an increase in enzymatic and non-enzymatic antioxidant will cause a decline in photosynthetic pigments (chlorophyll *a*, *b*, total chlorophyll, and carotenoids) which will directly cause a decline in morphological parameters and decline in yield. The MDA and H_2_O_2_ showed a strong negative correlation with all plant biomass attributes.

## Discussion

Globally, the saline soil is predicted to increase because of insufficient rainfall, highly-saline irrigating water, and poor agricultural management strategies, particularly in semi-arid and arid zones where evapotranspiration exceeds than precipitation [51]. In current study, it was observed that parameters related to growth, including the length of shoot and root, as well as the fresh and dry weights of shoot and root, markedly decreased under saline stress. Salinity hinders the development and growth of plant by impressive constraints. One primary mechanism contributing to these effects is osmotic stress is first constraint which reduces the plant ability for uptake of water. There are numerous events occurring in the plant but this is considering as main event in plant tissues that are under stress [52]. Lower water uptake immediately blocked the cell expansion that leads toward the loss in turgor pressure [53]. Exogenous SA led to enhanced growth parameters, including increased length of the root and shoot, greater shoot fresh and dry weight, and higher fresh and dry root weight. Salicylic acid helps to reduce the harmful effects of salinity by controlling photosynthesis through modulation of enzyme activity related to CO_2_ fixation and enhancement of stomatal conductance. It provides protection to plants cells against oxidative stress by managing both antioxidant systems i.e., enzymatic and non-enzymatic, and maintaining ion homeostasis by regulating the movement of H^+^ ions in plant roots [54, 55].

Both canola varieties exhibited a substantial reduction in photosynthesis-related pigments including chlorophyll *a*, chlorophyll *b*, total chlorophyll, and carotenoids under salt stress. The decrease was attributed to the breakdown of photosynthetic mechanism because chlorophyllase enzyme activity increased that interrupts the photosynthetic activity [56, 57]. All photosynthetic pigments significantly increased in both canola varieties by the application of SA. Exogenously applied SA plausibly restored the production of foliar photosynthetic pigments under salinity as the phytohormone improved homeostasis thereby giving cellular protection against salt-induced damage [58].

The concentration of reactive oxygen species i.e. MDA and H_2_O_2_ considerably increased under salt stress. The formation of different types of ROS (H_2_O_2_ and MDA) under salinity caused oxidative stress which violate the membrane system (both cellular and organelle) and influence lipid peroxidation. Numerous factors ionic and oxidative stress contribute to the generation and release of ROS that restricts photosynthetic activity and cause chlorophyll degradation [59]. SA considerably reduced H_2_O_2_ level by increasing antioxidant enzymes such as SOD, POD and CAT, which plays crucial role in ROS detoxification and maintenance of cellular redox homeostasis under both saline and control environment. Foliar applications of SA effectively reduced the increased levels of hydrogen peroxide (H_2_O_2_) and preserved cell membranes from oxidative harm. Salicylic acid achieved this by functioning as antioxidants, directly scavenging H_2_O_2_, and indirectly by stimulating the activity of antioxidant enzyme [60].

In current study, the concentration of enzymatic antioxidants such as CAT, POD, and SOD considerably enhanced under salt stress. The notable increase in enzymatic antioxidants is a plant’s adaptive response to counteract ROS-induced oxidative damage. The main scavenger of superoxide anion radical (O_2_^•.−^) is enzyme SOD and its action produces O_2_ and H_2_O_2_. Additional antioxidant enzymes like CAT and POD, then suppress the generated H_2_O_2_. Enzymatic antioxidants comprising POD and CAT detoxify ROS and metamorphose H_2_O_2_ into water and molecular oxygen [61, 62]. The enzymatic antioxidants, such as CAT, POD, and SOD considerably increased in both canola varieties by the application of foliar SA under both saline and control conditions. Salicylic acid performs important role in improving plant tolerance against stress by enhancing the antioxidative defense system [63]. It influences the activity of antioxidant enzymes by decreasing the destructive effects of ROS under saline stress [64, 65]. Foliar applications of SA efficiently diminish the high levels of H_2_O_2_ and protect cell membranes from oxidative destruction. Because of its distinctive properties, SA prevents membrane oxidation loss by acting directly as an antioxidant to scavenge H_2_O_2_ and indirectly by activating antioxidant enzymes [66, 67].

The amount of TSP, as well as TSS, and endogenous AsA significantly increased in both varieties under saline conditions. The accumulation of osmolytes such as TSS, TSP and endogenous AsA are one of the key physiological indicators of salt tolerance in plants, which is considered an essential mechanism employed by many plants to cope with salt stress [68, 69]. These compounds under salt stress protects cells by balancing the osmotic potential of the cytosol with that of the vacuole [70]. Salicylic acid considerably increases TSS, TSP and endogenous AsA under both salinity and control conditions. SA enhanced the salinity tolerance by osmotic adjustment through maintaining membrane stability and preserving enzyme activity involved in osmolyte metabolism that are essential mechanisms which enable plants to safeguard their tissues from damage thereby enabling uninterrupted growth and development in saline environments [22, 71].

Secondary metabolites such as total phenolics, anthocyanin and flavonoids increased under salt stress. These compounds are known for their key function in preventing salt stress, which can lead to oxidative damage in plants [72]. Phenolic compounds demonstrate antioxidant properties by neutralizing free lipid radicals and preventing the conversion of hydro peroxides into free radicals. This enhanced antioxidant activity aids in the detoxification of ROS, likely contributing to increased resistance against salinity [73, 74]. Secondary metabolites such as phenols, anthocyanin, and flavonoids considerably increased by the exogenous use of SA under control and salt stress in current experiment. SA acts as a signaling molecule that triggers various defense mechanism in plants. One of these mechanisms involves maintaining membrane integrity and enzymatic action. These secondary metabolites assist the plant in avoiding tissue damage, scavenge harmful ROS thus improving the plant defense system from various harmful effects of salinity allowing for continuing development and progress under challenging circumstances [75].

Our study demonstrated that under salt stress, proline and glycine betaine increased in both varieties of canola. The elevation of these compatible solutes and osmoprotectant under salt stress reflects the plant’s adaptive response to counteract salinity’s adverse effects. Proline is essential for maintaining the stability of membranes because it binds to membrane phospholipids, which alters the hydrated layer around biological macromolecules and aids in safeguarding cellular structures against the disruptive effects of salt stress. Use of exogenous SA improved the production of glycine betaine and proline in varieties both saline and non-saline environments [76]. Glycine betaine and proline have ability to scavenge ROS production and resist salt stress. Glycine betaine and proline have a potential to assist the plant in preventing tissue damage as these two inhibit the production of destructive ROS which contributes to the plant defense system from various harmful effects of salinity and allowing continued growth and development under stressful conditions [77, 78].

The substantial increase in root and shoot Na^+^ and a considerable decrease in potassium (K^+^) and calcium (Ca^2+^) ion under salinity was observed. The excessive buildup of sodium (Na^+^) can be detrimental to various aspects of plant health [79]. It disrupts the balance of water and nutrient uptake, impairs metabolic processes, disturbs ionic equilibrium, and hinders crucial plant developmental processes, ultimately leading to plant death [80]. Potassium and calcium play dynamic osmoregulation functions, enzymes activation and cytoplasmic homeostasis maintenance [36]. Application of SA cause significant reduction in root and shoot sodium ions (Na^+^) and increased the K^+^ and Ca^2+^ ions. Maintaining proper ion balance and cellular homeostasis is crucial for plants to effectively cope with salt stress. Plants treated with SA showed a significant decrease in sodium (Na^+^) concentration and a notable increase in K^+^ uptake. This could be attributed to the combined influence of both compounds in regulating nutrient uptake and maintaining ionic stability in plants [81].

## Conclusion

Under high salinity conditions, both canola varieties experienced a decline in morpho-physiological and biochemical attributes, including a notable reduction in photosynthetic pigments and the accumulation of ROS. Interestingly, the adverse effects of salt stress were mitigated by the application of SA as a foliar spray. Salicylic acid treatment (10 mM and 20 mM) played a crucial role in maintaining osmotic balance, facilitating nutritional absorption and mineral ion uptake, and aiding in ROS detoxification by promoting the production of enzymatic and non-enzymatic antioxidants and osmolytes. The efficacy of SA in alleviating the effects of salt stress has been well established. Additionally, it was observed that variety V2 (Super canola) demonstrated greater resilience to salinity stress compared to variety V1 (Faisal canola), as evidenced by its lesser deterioration in morphological features, antioxidant metabolism, and nutritional absorption when subjected to SA application. Further investigations should be conducted to explore the application of SA using different methods in field settings on a larger scale.

## Data Availability

All data generated or analyzed during this study are included in this published article.
